# A new species of *Pseudasphondylia* (Diptera: Cecidomyiidae) associated with *Magnolia kobus* DC. var. *borealis* Sarg. (Magnoliaceae) in Japan

**DOI:** 10.3897/BDJ.9.e68016

**Published:** 2021-06-17

**Authors:** Hiroki Matsuda, Ayman Khamis Elsayed, Wanggyu Kim, Satoshi Yamauchi, Martin Libra, Naoto Kamata, Junichi Yukawa, Makoto Tokuda

**Affiliations:** 1 Laboratory of Systems Ecology, Faculty of Agriculture, Saga University, Saga, Japan Laboratory of Systems Ecology, Faculty of Agriculture, Saga University Saga Japan; 2 IDEA consultants, inc., Yokohama, Japan IDEA consultants, inc. Yokohama Japan; 3 The Botanical Gardens, Graduate School of Science, The University of Tokyo, Tokyo, Japan The Botanical Gardens, Graduate School of Science, The University of Tokyo Tokyo Japan; 4 Animal Resources Division, National Institute of Biological Resources, Incheon, Republic of Korea Animal Resources Division, National Institute of Biological Resources Incheon Republic of Korea; 5 Shinjo, Aomori, Japan Shinjo Aomori Japan; 6 Faculty of Science, University of South Bohemia, Ceske Budejovice, Czech Republic Faculty of Science, University of South Bohemia Ceske Budejovice Czech Republic; 7 Biology Centre of the Czech Academy of Sciences, Institute of Entomology, Ceske Budejovice, Czech Republic Biology Centre of the Czech Academy of Sciences, Institute of Entomology Ceske Budejovice Czech Republic; 8 The University of Tokyo Hokkaido Forest, Graduate School of Agricultural and Life Sciences, The University of Tokyo, Furano, Japan The University of Tokyo Hokkaido Forest, Graduate School of Agricultural and Life Sciences, The University of Tokyo Furano Japan; 9 Entomological Laboratory, Faculty of Agriculture, Kyushu University, Fukuoka, Japan Entomological Laboratory, Faculty of Agriculture, Kyushu University Fukuoka Japan; 10 The United Graduate School of Agricultural Sciences, Kagoshima University, Kagoshima, Japan The United Graduate School of Agricultural Sciences, Kagoshima University Kagoshima Japan

**Keywords:** Asphondyliini, Asphondyliina, gall midge, molecular phylogeny

## Abstract

**Background:**

A gall midge species (Diptera: Cecidomyiidae) inducing leaf bud galls on Magnolia kobus DC. var. borealis Sarg. (Magnoliaceae) was found in Hokkaido and northern Honshu, Japan.

**New information:**

Based on its morphology, the species is regarded as an undescribed species of the genus *Pseudasphondylia* Monzen (Cecidomyiinae, Cecidomyiidi, Asphondyliini). The species is herein described as *Pseudasphondylia saohimea* Matsuda, Elsayed and Tokuda **sp. n.** The new species is easily distinguishable from its congeners by the number of adult palpal segments and the shape of the male terminalia and larval spatula.

## Introduction

The genus *Pseudasphondylia* (Diptera: Cecidomyiidae: Asphondyliini) includes 11 species associated with various plant families (Gagné and Jaschhof 2021). Nine of them are distributed in the eastern Palearctic Region (e.g. Elsayed et al. 2019, Tokuda and Yukawa 2005), one in India and one in New Caledonia (Gagné and Jaschhof 2021). Amongst the nine eastern Palaearctic species, the following six species have been recorded from Japan: *P. elaeocarpi* Tokuda and Yukawa on *Elaeocarpus zollingeri* K.Koch (= E. sylvestris var. ellipticus Hara) (Elaeocarpaceae), *P. kiritanii* Tokuda & Yukawa on *Cornus controversa* Hemsl. ex Prain (Cornaceae), *P. matatabi* (Yuasa & Kumazawa) on *Actinidia polygama* (Siebold et Zucc.) Planch. ex Maxim. (Actinidiaceae), *P. neolitseae* Yukawa on *Neolitsea sericea* (Blume) Koidz. (Lauraceae), *P. rokuharensis* Monzen on *Viburnum dilatatum* Thunb. (Caprifoliaceae) and *P. tominagai* Elsayed & Tokuda on *Eleutherococcus spinosus* (L.f.) S.Y.Hu (Araliaceae) (Monzen 1955, Tokuda 2012, Tokuda and Yukawa 2002, Tokuda et al. 2007, Yukawa 1974, Yukawa and Masuda 1996, Elsayed et al. 2019, Yukawa 1983). They have type IIA or IIB life history strategy, in which mature (type IIA) or immature (type IIB) larvae overwinter in galls on their host plants (Yukawa 1974, Yukawa 1987, Tokuda and Yukawa 2005, Tokuda et al. 2007). In addition, some species exhibit polymodal emergence patterns caused by long-term diapause (Takasu and Yukawa 1984) and other species are suspected to alternate host plants (Yukawa and Masuda 1996, Tokuda and Yukawa 2005, Elsayed et al. 2019a).

In recent years, we found an undescribed species of asphondyliine gall midge that induces leaf bud galls on Magnolia kobus DC. var. borealis Sarg. (Magnoliaceae) in Tomakomai, Hokkaido and Aomori Prefecture, Honshu, Japan. Based on morphological comparison, we concluded that the gall midge is a member of *Pseudasphondylia*. We describe the species as new to science and discuss the phylogenetic relationships amongst Japanese congeners on the basis of molecular analysis. We also compare its life history strategy with those of the Japanese congeners.

## Materials and methods

### Collecting and Rearing

**Figure 1.**
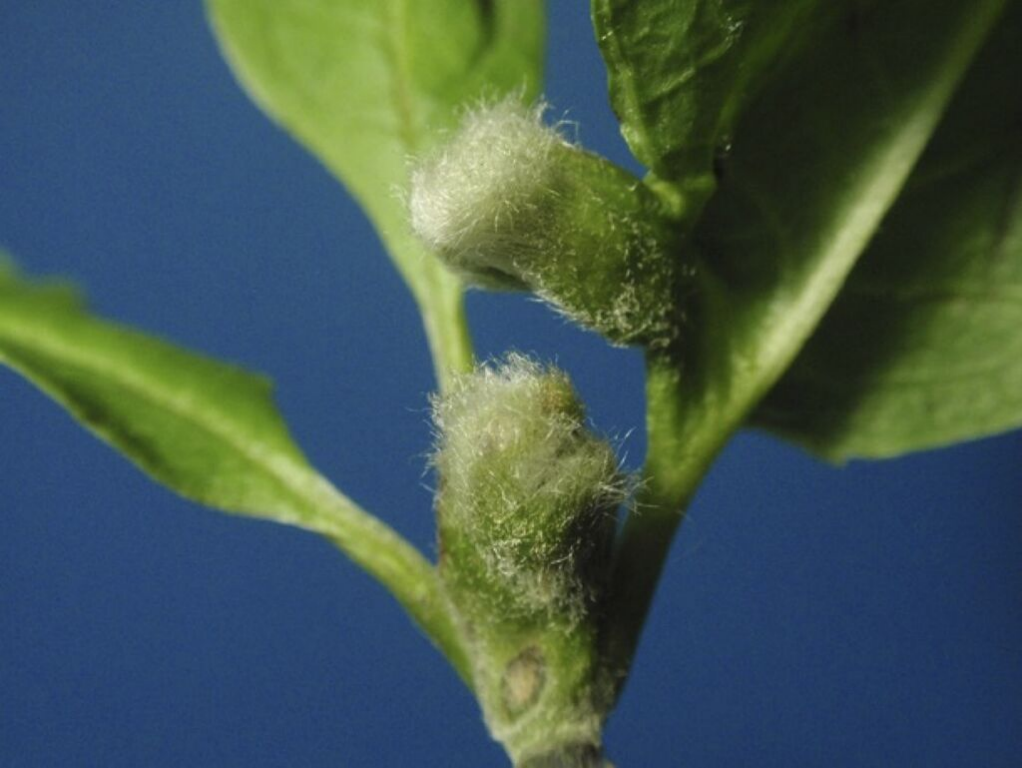
Leaf galls induced by *Pseudasphondylia sahohimea* **sp. n.** on Magnolia kobus var. borealis.

Leaf-bud galls on M. kobus var. borealis (Fig. 1) were collected from Tomakomai City, Hokkaido and Mutsu City, Aomori Prefecture, Honshu, Japan. Some galls were dissected under a stereoscopic microscope to obtain mature larvae and others were kept in plastic bags until emergence of adults. Most specimens were preserved either in 75% ethanol for morphological examinations or in 99.5% ethanol for molecular analyses.

### Morphological Examination and Terminology

Gall midge specimens were mounted on slides in Canada balsam, following the technique outlined in Gagné (1994), except for the clearing step for the larval and adult specimens following Elsayed et al. (2018). The slide-mounted specimens were examined under a bright-field and phase-contrast microscope (H550L, Nikon, Tokyo) and line illustrations were made following Elsayed et al. (2020) under a bright-field and phase-contrast microscope (CX43, Olympus, Tokyo). Photomicrographs were taken with a digital camera (DP22, Olympus, Tokyo) attached to a semi-motorised fluorescence microscope (BX53, Olympus, Tokyo).

Morphological terminology basically follows Gagné (2018), except the term “antennal papillae” in pupa follows Elsayed et al. (2020a). Larval and pupal terminology follow Gagné (1994). The holotype and paratypes are deposited in the collection of the Entomological Laboratory, Faculty of Agriculture, Kyushu University, Japan (ELKU). A generic synopsis of *Pseudasphondylia* is presented in Tokuda and Yukawa (2005).

### Molecular Phylogenetic Analysis

Genome DNA was extracted from 99.5% ethanol-preserved female gall midges reared from leaf galls of M. kobus var. borealis and of *P. tominagai* reared from flower bud galls of *E. spinosus* (Elsayed et al. 2019) and from adults of *P. elaeocarpi* obtained from leaf galls on E. sylvestris var. ellipticus (Elaeocarpaceae). A 472 bp fragment of the mitochondrial gene cytochrome oxidase subunit I (COI) of the species from leaf galls of M. kobus var. borealis and *P. tominagai* was sequenced and aligned following Elsayed et al. (2017), using the following primer set: Forward: J-1718 (5’–GGA GGA TTT GGA AAT TGA TTA GTT CC–3’) (Simon et al. 1994) and reverse: COIA (5’–CCC GGT AAA ATT AAA ATA TAA ACT TC–3’) (Funk et al. 1995), while a 439 bp fragment of the same gene of *P. elaeocarpi* was sequenced using the primer set: COIS (5’–GGA TCA CCT GAT ATA GCA TTC CCA TAT TGG–3’) and COIA (5'–CCC GGT AAA ATT AAA ATA TAA ACT TC–3’) (Funk et al. 1995). The sequences obtained were compared using MEGA (version X) (Kumar et al. 2018) and deposited in the DNA Data Bank of Japan (DDBJ), European Molecular Biology Laboratory (EMBL) and GenBank nucleotide sequence databases as accession numbers LC621302–621303, LC621304–621305 and LC621306 for *P. tominagai*, the gall midge of M. kobus var. borealis and *P. elaeocarpi*, respectively.

Besides the obtained sequence data, sequences of four species of *Pseudasphondylia* and four species of *Asphondylia* were downloaded from the GenBank and used as ingroup taxa: *P. rokuharensis* (LC538357), *P. kiritanii* (LC538356), *P. matatabi* (AB085873) (Lin et al. 2020), *P. neolitseae* (AB334237) (Tokuda et al. 2008), *Asphondylia tojoi* Elsayed and Tokuda (LC373200) (Elsayed et al. 2018b), *A. aucubae* Yukawa & Ohsaki (AB238595) (Uechi and Yukawa 2006b), *A. yushimai* Yukawa and Uechi (AB194473) (Uechi et al. 2005) and *A. sphaera* Monzen (AB197945) (Uechi and Yukawa 2006a). GenBank sequences of two distant species were used as outgroup taxa: *Ampelomyia conicocoricis* Elsayed and Tokuda (LC422091) (Elsayed et al. 2019b) and *Gephyraulus zewaili* Elsayed and Tokuda (LC270942) (Elsayed et al. 2017a). These sequences were aligned using ClustalW algorithm in MEGA X. We inferred the phylogenetic relationships in the same software using the Maximum Likelihood (ML) method and 1000 bootstrap replications, based on the GTR+I model determined by jModelTest 2 (Darriba et al. 2012, Guindon and Gascuel 2003).

## Taxon treatments

### Pseudasphondylia saohimea

Matsuda, Elsayed & Tokuda sp. n.

34B09DDF-9268-5078-8309-13576CB34083

C7679EFB-7E8C-4157-A5AE-C618D2D82B28

#### Materials

- **Type status:** Holotype. **Occurrence:** sex: male; lifeStage: adult; **Taxon:** order: Diptera; family: Cecidomyiidae; genus: Pseudasphondylia; specificEpithet: *saohimea*; taxonRank: species; nomenclaturalCode: ICZN; **Location:** country: Japan; stateProvince: Aomori; locality: Ashizaki, Mutsu City; **Event:** samplingProtocol: reared from a leaf bud gall on Magnolia kobus var. borealis (Magnoliaceae) collected on 10.v.2015 by S. Yamauchi
- **Type status:** Paratype. **Occurrence:** individualCount: 7; sex: males; lifeStage: adult; **Taxon:** order: Diptera; family: Cecidomyiidae; genus: Pseudasphondylia; specificEpithet: *saohimea*; taxonRank: species; nomenclaturalCode: ICZN; **Location:** country: Japan; stateProvince: Aomori; locality: Ashizaki, Mutsu City; **Event:** samplingProtocol: reared from leaf bud galls on Magnolia kobus var. borealis (Magnoliaceae) collected on 10.v.2015 by S. Yamauchi
- **Type status:** Paratype. **Occurrence:** individualCount: 8; sex: females; lifeStage: adult; **Taxon:** order: Diptera; family: Cecidomyiidae; genus: Pseudasphondylia; specificEpithet: *saohimea*; taxonRank: species; nomenclaturalCode: ICZN; **Location:** country: Japan; stateProvince: Aomori; locality: Ashizaki, Mutsu City; **Event:** samplingProtocol: reared from leaf bud galls on Magnolia kobus var. borealis (Magnoliaceae) collected on 10.v.2015 by S. Yamauchi
- **Type status:** Paratype. **Occurrence:** individualCount: 3; lifeStage: mature larvae; **Taxon:** order: Diptera; family: Cecidomyiidae; genus: Pseudasphondylia; specificEpithet: *saohimea*; taxonRank: species; nomenclaturalCode: ICZN; **Location:** country: Japan; stateProvince: Aomori; locality: Ashizaki, Mutsu City; **Event:** samplingProtocol: collected on 19.v.2019 by S. Yamauchi at the type locality
- **Type status:** Paratype. **Occurrence:** individualCount: 4; lifeStage: pupal exuviae; **Taxon:** order: Diptera; family: Cecidomyiidae; genus: Pseudasphondylia; specificEpithet: *saohimea*; nomenclaturalCode: ICZN; **Location:** country: Japan; stateProvince: Aomori; locality: Ashizaki, Mutsu City; **Event:** samplingProtocol: reared from a leaf gall on Magnolia kobus var. borealis (Magnoliaceae) collected on 10.v.2015 by S. Yamauchi
- **Type status:** Paratype. **Occurrence:** individualCount: 3; sex: females; lifeStage: adult; **Taxon:** order: Diptera; family: Cecidomyiidae; genus: Pseudasphondylia; specificEpithet: *saohimea*; taxonRank: species; nomenclaturalCode: ICZN; **Location:** country: Japan; stateProvince: Hokkaido; locality: Takaoka (Tomakomai Experimental Forest, The Field Science Center for Northern Biosphere, Hokkaido University), Tomakomai City; **Event:** samplingProtocol: collected on 31.v.2015 by M. Libra
- **Type status:** Paratype. **Occurrence:** individualCount: 1; sex: male; lifeStage: adult; **Taxon:** order: Diptera; family: Cecidomyiidae; genus: Pseudasphondylia; specificEpithet: *saohimea*; taxonRank: species; nomenclaturalCode: ICZN; **Location:** country: Japan; stateProvince: Hokkaido; locality: Takaoka (Tomakomai Experimental Forest, The Field Science Center for Northern Biosphere, Hokkaido University), Tomakomai City; **Event:** samplingProtocol: collected on 31.v.2015 by M. Libra

#### Description

**Figure 2.**
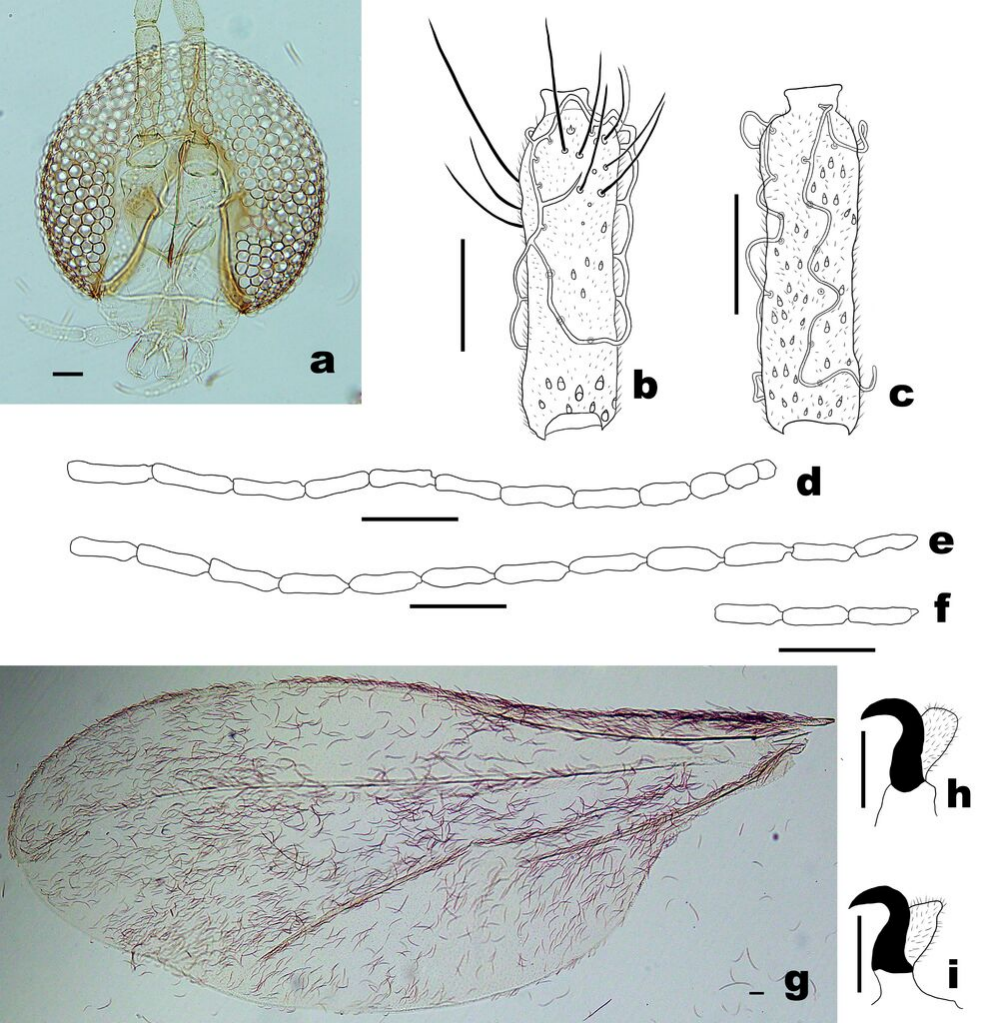
*Pseudasphondylia saohimea* **sp. n. a.** Head; **b.** Female flagellomere V. **c.** Male flagellomere V; **d.** General shape of female flagellomeres; **e.** General shape of male flagellomeres; **f.** Male flagellomeres X–XII; **g.** Wing; **h.** Acromere of fore-leg. **i.** Acromere of hind-leg. Scale bars = 50 µm.

**Head** (Fig. 2a-f). Eye bridge 6–7 facets long, facets rounded. Antenna: scape with more setae ventrally than dorsally; pedicel spheroid, with few scattered setae ventrally and dorsally; flagellomeres generally cylindrical, nodes setose and microtrichose, with appressed circumfila and short, naked necks; male flagellomeres I–II not fused, female flagellomeres I–IX becoming noticeably shorter successively, flagellomeres X–XII successively more foreshortened, flagellomere X 1.5 times as long as wide, flagellomere XI about 1.3 times as long as wide, flagellomere XII spheroid; male flagellomeres with anastomosing wavy circumfila; male flagellomere XII sometimes with tiny apical projection as in Fig. 2f. Palpus: 3–segmented, each with a few setae and scales, first segment shortest, 23–28 μm long, second about twice as long as first, third about twice as long as second.

**Thorax** (Fig. 2g-i). Anepisternum with 23–26 scales; anepimeron with 17–23 setae (n = 6). Acropods: claws bent after mid-length, empodia as long as claws. Wing: length 2.5–3.2 mm (n = 4) in male and 2.9–3.6 mm (n = 4) in female; width 1.0–1.4 mm (n = 4) in male and 1.2–1.6 mm (n = 4) in female; Rs joining C posterior to wing apex.

**Figure 3.**
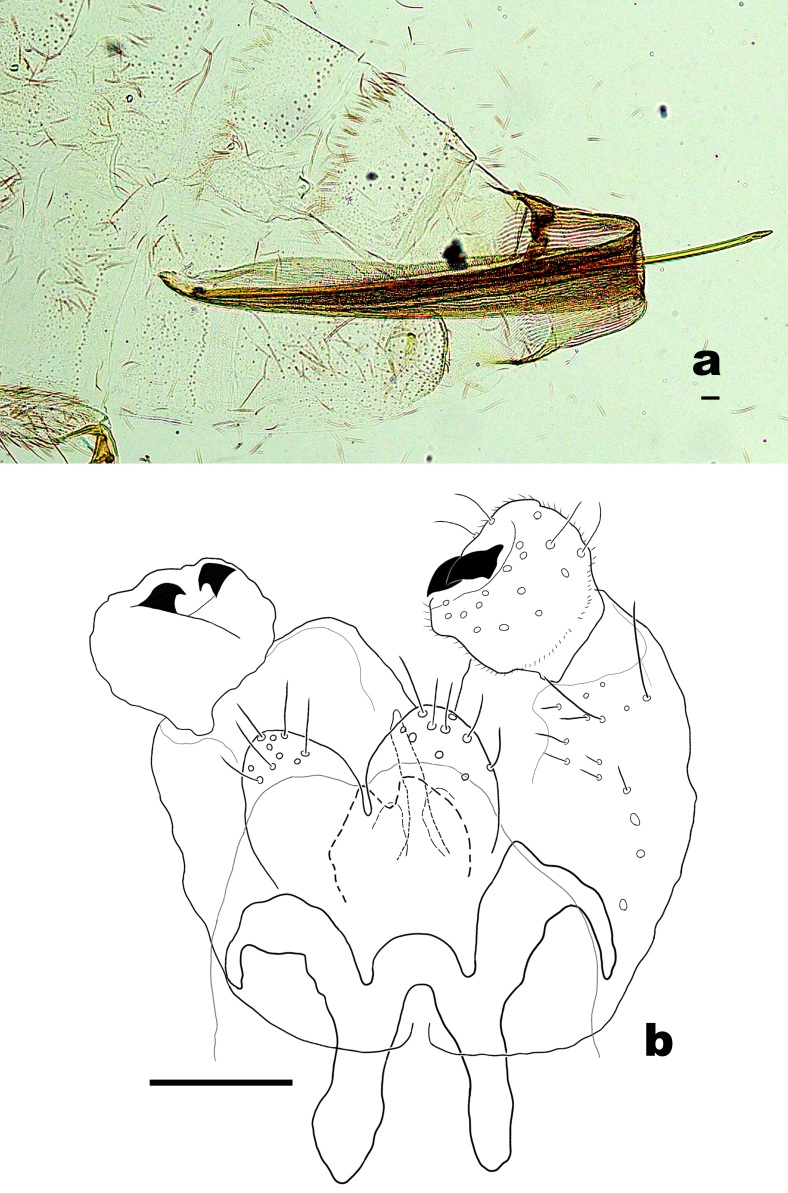
*Pseudasphondylia saohimea* **sp. n. a.** Terminal part of female abdomen; **b.** Male terminalia. Scale bars = 50 µm.

**Female abdomen** (Fig. 3a). Tergites I–VII rectangular, evenly covered with scales, with lateral setae and without anterior pair of trichoid sensilla; tergites I–VI with 1-2 posterior rows of setae, but tergite VII with 2-3 rows; tergite VIII bare. Sternites II–VII with anterior pair of trichoid sensilla situated laterally; sternites II–VI rectangular, anteriorly with scattered setae and scales, posteriorly with one row of setae usually mixed with some scales; sternite VII about two times longer than VI, covered with scattered setae and scales. Ovipositor: protrusible needle-like portion about 3.5 (3.4–3.6; n = 3) times longer than sternite VII; cerci undifferentiated.

**Male abdomen** (Fig. 3b). Tergites I–VII with 2-3 posterior rows of setae, otherwise as in female. Sternites II–VII as sternites II–VI in female; sternite VIII about 0.5 times shorter than VII, covered with scattered setae and few scales. Terminalia (Fig. 3b): Gonostylus suboval, with setae dorsally and ventrally on distal two thirds, with two sclerotised teeth; cerci oval, setose; hypoproct shorter than cerci, basally wider than distally, bilobed, each lobe with one seta; gonocoxal lobes present; aedeagus tapered.

**Figure 4.**
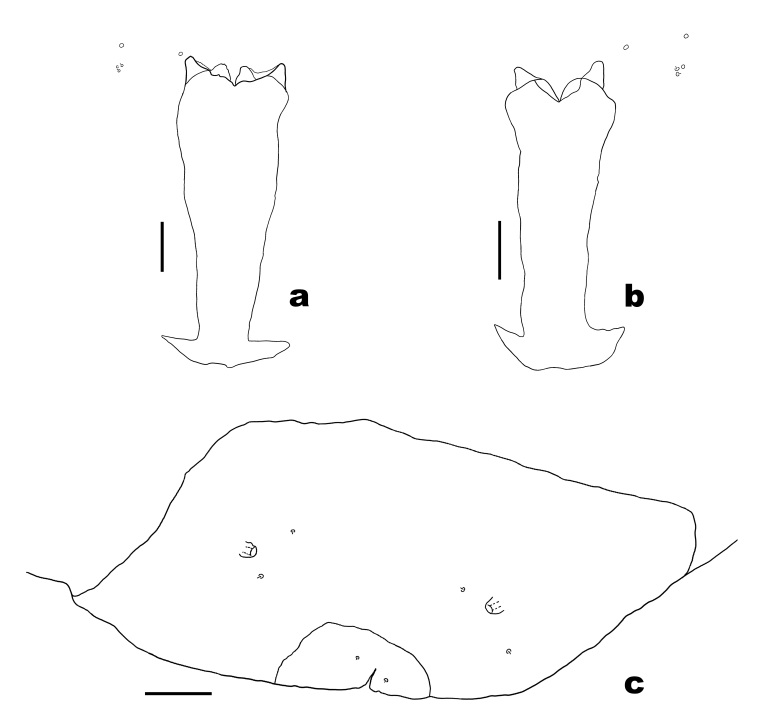
Larva of *P. saohimea* **sp. n. a-b.** Shape variation in sternal spatula and associated papillae; **c.** Dorsal view of 8^th^ and terminal abdominal segments. Scale bars = 50 µm.

**Mature larva** (Fig. 4). Body colour in life orange. Spatula: anteriorly with four lobes, outer two longer than inner two. Three lateral papillae present on each side of midline, two with setae. Three pairs of asetose pleural papillae present anteriorly on prothorax. Two pairs of asetose pleural papillae on meso- and metathorax. One pair of setose pleural papillae on abdominal segments I–VIII. Two sternal papillae on each thoracic segment and abdominal segments I–VII, with setae, except on prothorax without setae. Two pairs of dorsal papillae present, without setae on thoracic segments and only outer pair with setae on abdominal segments I–VII; a pair of setose dorsal papillae on abdominal segment VIII. Terminal abdominal segment with two setose terminal papillae and two asetose anal papillae present.

**Figure 5.**
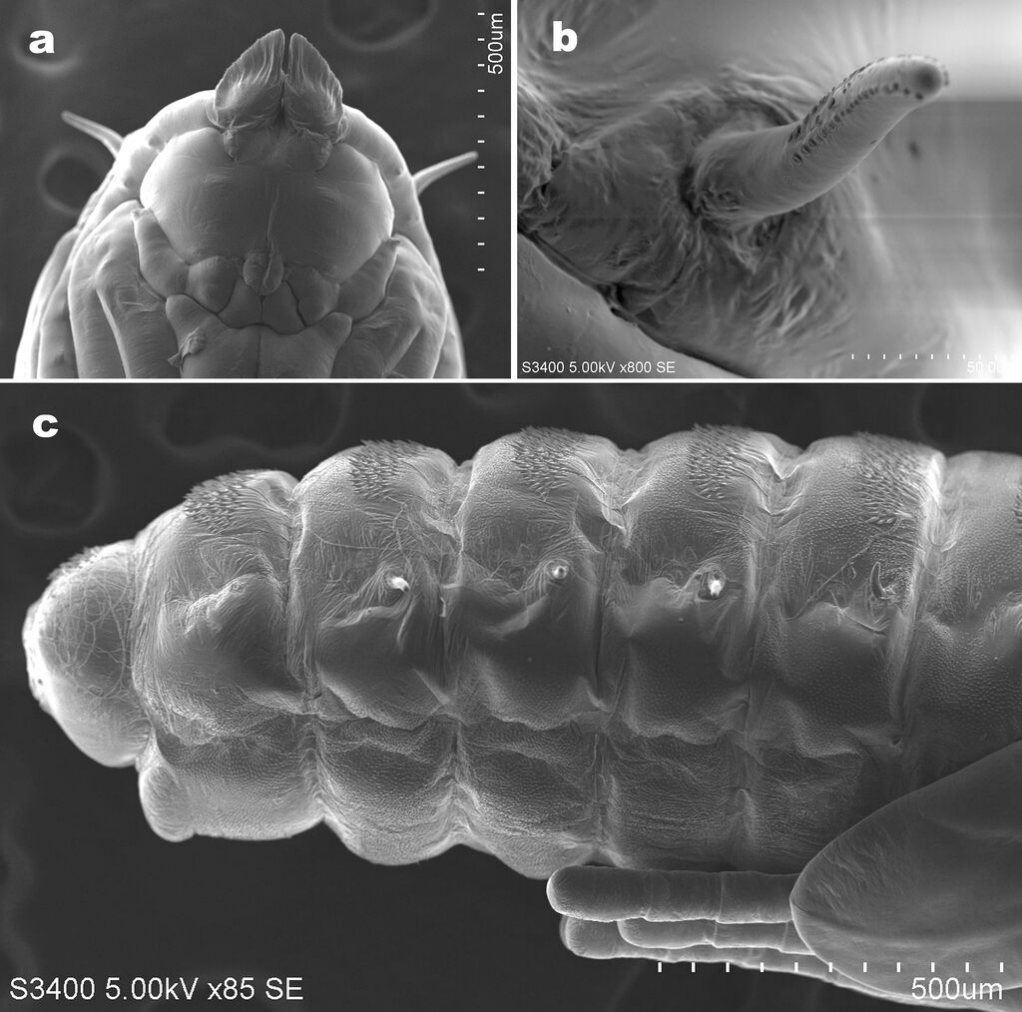
Scanning electron micrographs of pupa of *Pseudasphondylia saohimea* **sp. n. a.** Ventral view of head **b.** Prothoracic spiracle; **c.** Lateral view of abdominal segments. Scale bars = 50 µm.

**Pupa** (Fig. 5). Two setose and two asetose cephalic papillae on tubercles. Antennal horns greatly enlarged, tapered and dorsoventrally flattened, serrate along anterior margin. Antennal papillae absent. Lower and lateral facial papillae not visible. Prothoracic spiracle elongated, slightly curved, about 160 μm long, with tracheae extending to tip. Abdominal spiracles present on segments II–VI, each spiracle about 0.3 times as long as prothoracic spiracle. Abdominal terga I–VII with anterior pair of trichoid sensilla, 5–6 rows of spines and three pairs of setose dorsal papillae; tergum VIII with 5–6 rows of spines and two setose dorsal papillae.

#### Etymology

The specific name, *saohimea*, is derived from “Saohime”, a Japanese goddess of spring, because blooming of the host plant Magnolia kobus var. borealis is a symbolic event announcing the beginning of spring in northern Japan. Galls of *P. saohimea* become conspicuous on the host also in early spring.

#### Distribution

Japan, Hokkaido and Honshu (Aomori Prefecture).

#### Biology

*Pseudasphondylia saohimea* is univoltine. Third instars and pupae were found in the galls in mid-May and adults emerged directly from the galls in mid- to late May. All mature galls collected in July were empty, indicating that no individuals had entered long-term diapause. In rearing conditions, adults emerged in the morning and mated around 11:00 h, suggesting that the gall midge is a diurnal species. The adults are supposed to oviposit into host buds. First instars were found in undeveloped bud galls in late September. They possibly overwinter in the undeveloped bud galls and develop to the second and third instars in the following spring.

##### Host plant

Magnolia kobus DC. var. borealis Sarg. (Magnoliaceae), “Kita-kobushi” in Japanese.

##### Gall

*Pseudasphondylia saohimea* induces hairy leaf bud galls on Magnolia kobus var. borealis (Magnoliaceae). The galled buds remain closed and indistinguishable in appearance from ungalled buds until the following spring and rapidly grow with bud burst. Mature galls are 2.7–6.0 mm in diameter and 5.1–13.7 mm in length (n = 45). Galls are multi-chambered and each chamber contains a single gall midge larva.

##### Parasitoids

The following three species of hymenopteran parasitoids were reared from the mature galls: *Pseudocatolaccus* sp. (Pteromalidae) from Hokkaido and Aomori, *Torymus* sp. (Torymidae) from Hokkaido and *Eurytoma* sp. (Eurytomidae) from Aomori.

#### Notes

The new species is distinguishable from most of its other congeners in Japan by the number of palpal segments: three in the new species, but two in *P. neolitseae* and four in *P. rokuharensis*, *P. kiritanii* and *P. tominagai*. Although *P. matatabi* and *P. elaeocarpi* have three-segmented palpi, they are easily distinguished from the new species by their cerci which are shorter than the hypoproct. The larval spatula of the new species has four lobes of which the outer two are longer than the inner two. However, the larval spatula of *P. neolitseae* has only two lobes anteriorly and the other species have four lobes of which the inner two are longer than the outer two. In the pupa, five pairs of long abdominal spiracles are present in the new species, while only three pairs are present in *P. rokuharensis*, *P. kiritanii*, *P. elaeocarpi* and *P. tominagai* and four pairs in *P. matatabi*. The pupa of *P. neolitseae*, which has five pairs of abdominal spiracles, is otherwise similar to the new species, but it can be distinguished by dorsal abdominal spines that are markedly shorter than in *P. saohimea*.

In the key to the males of world *Pseudasphondylia* species in Elsayed et al. (2019), *P. saohimea* will run to couplet 5 that separates *P. matatabi* and *P. elaeocarpi*. In order to update the key and include *P. saohimea*, couplet 5 is amended and a sub-couplet is added as in Table 1.

##### Molecular phylogenetic analysis

**Figure 6.**
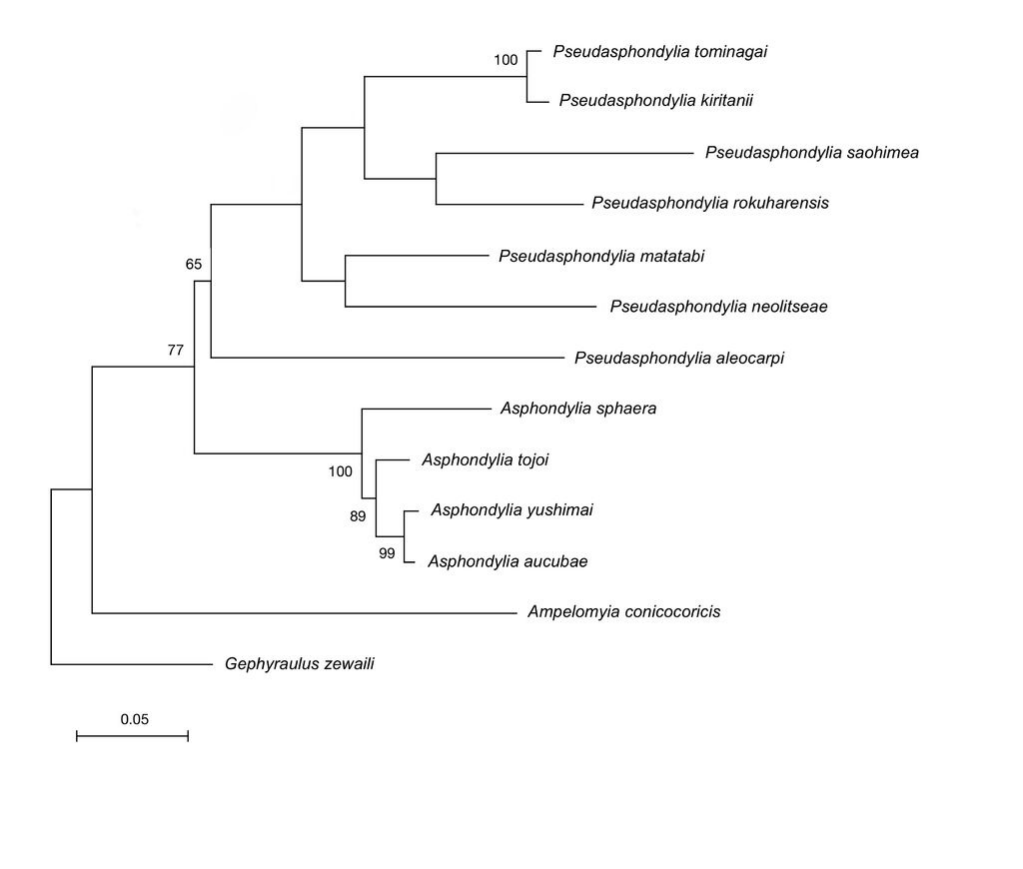
Maximum Likelihood phylogenetic tree of Japanese *Pseudasphondylia* species, based on a fragment of COI. Bootstrap values are indicated for nodes that gained > 50% support in 1,000 replications.

In the ML tree (Fig. 6), *Pseudasphondylia* species constructed a monophyletic clade relatively supported by 65% bootstrap value. Although *P. saohimea* constructed a clade with *P. rokuharensis*, their bootstrap support was lower than 50%. Genetic divergence of *P. saohimea* and the other Japanese *Pseudasphondylia* species was high and ranging between 15% to 21% (15% between *P. saohimea* and *P. rokuharensis*; 16% between *P. saohimea* and *P. matatabi*; 17% between *P. saohimea* and *P. tominagai* and *P. kiritanii*; 21% between *P. saohimea* and *P. neolitseae*).

## Discussion

Morphological examination and phylogenetic analysis clearly indicate that *P. saohimea* is distinct from its congeners in Japan, although phylogenetic relationships amongst *Pseudasphondylia* species were not revealed in the analyses, except for the sister group relationship of *P. kiritanii* and *P. tominagai*. *Pseudasphondylia* was hypothesised to be an ancient clade within the subtribe Asphondyliina (Tokuda and Yukawa 2007, Tokuda 2012), which suggests that it would be more promising to analyse more conservative DNA regions to illuminate the inner generic relationships.

Based on overwintering sites and larval stages, the life-history of gall midges is divided into two main types, of which each can be subdivided into two subtypes regardless of uni-, bi- or multivoltine (Yukawa 1987, Yukawa and Uechi 2021). Adaptive significance of respective life history strategies has been attributed particularly to the avoidance of parasitoid attack (Yukawa 1987, Tokuda et al. 2004, Yukawa and Rohfritsch 2005, Yukawa and Uechi 2021). Most species of Asphondyliini adopt type IIA or IIB life history strategy. Mature larvae overwinter in the galls on host plants in type IIA, while young larvae do so in type IIB. Amongst the six Japanese species of *Pseudasphondylia*, *P. neolitseae* and *P. rokuharensis* are univoltine-type IIA species, *P. elaeocarpini* is a univoltine-type IIB species and *P. kiritanii*, *P. matatabi* and *P. tominagai* are host-alternating, bivoltine and possibly type IIB species (Yukawa 1974, Yukawa and Masuda 1996, Tokuda and Yukawa 2005, Tokuda et al. 2007, Elsayed et al. 2019a).

As mentioned above, *P. saohimea* belongs to the univoltine type IIB, as does *P. elaeocarpi* (Tokuda and Yukawa 2005). However, leaf galls induced by *P. elaeocarpi* become full-grown in summer and the first instars spend summer, autumn and winter in fully developed galls (Yukawa and Masuda 1996, Tokuda and Yukawa 2005). This contrasts with the situation in *P. saohimea*, which passes through these seasons in inconspicuous galls.

Some Asphondyliini, other than *Pseudasphondylia*, have a life history pattern similar to that of *P. saohimea*. For example, females of *Oxycephalomyia styraci* (Shinji) oviposit into axillary overwintering buds of *Styrax japonica* Siebold and Zucc. (Styracaceae) and its larvae spend summer, autumn and winter as the first instar in the host buds; then, galls and larvae rapidly grow, coinciding with host bud burst (Tokuda et al. 2004). Tokuda et al. (2004) proposed that the life history of *O. styraci* is adaptive to avoid late parasitoids, which are generally idiobiont ectoparasitoids attacking mature host larvae. Similarly, *Asphondylia tojoi* Elsayed and Tokuda, inducing unremarkable leaf bud galls on *Schoepfia jasminodora* Siebold and Zucc. (Schoepfiaceae), overwinters as the first instar in inconspicuous overwintering buds, although this species is multivoltine and repeatedly uses axillary buds throughout the year (Elsayed et al. 2018b).

Some large genera, such as *Asphondylia*, *Contarinia*, *Dasineura* and *Lasioptera*, are associated with various plant families, while others are restricted to particular plant genera, namely *Caryomyia* on *Carya* (Juglandaceae), *Procontarinia* on *Mangifera* (Anacardiaceae), *Rabdophaga* on *Salix* (Salicaceae) and *Semudobia* on *Betula* (Betulaceae) (Yukawa et al. 2005). *Pseudasphondylia* belongs to the former group, although it, so far, contains only a small number of species. Considering the antiquity of *Pseudasphondylia* and its wide host range and diverse life history patterns, many more species may remain undiscovered, since generally, in Cecidomyiidae, ecological diversifications are involved in radiation at the species level (Yukawa et al. 2019).

## Supplementary Material

XML Treatment for Pseudasphondylia saohimea

## Figures and Tables

**Table 1.** Placement of *Pseudasphondylia saohimea* in the key to males of worldwide *Pseudasphondylia* (Elsayed et al. 2019a)

5	Tergites I–VII with 2–3 posterior rows of setae	5’
	Tergites I–VII with 1 posterior row of setae	*P. elaeocarpi* Tokuda & Yukawa
5'	Cerci shallowly separated (Tokuda and Yukawa 2005); wing length approximately 2.2 times as long as wide (Fig. 3e in Elsayed et al. 2019a)	*P. matatabi* (Yukawa & Kumazawa)
	Cerci deeply separated, each cercus oval (Fig. 3b); wing length approximately 2.4 times as long as wide (Fig. 2g)	*P. saohimea* **sp. n.**
